# Novel anatomical-based surgical technique for positioning of the patellar component in total knee arthroplasty

**DOI:** 10.1051/sicotj/2017053

**Published:** 2017-12-11

**Authors:** Chahine Assi, Nadim Kheir, Camille Samaha, Moussa Chamoun, Kaissar Yammine

**Affiliations:** 1 Lebanese American University Medical Center-Rizk Hospital, Department of Orthopedic Surgery, Beirut Lebanon; 2 Hopital Libano-Français, Zahle Lebanon; 3 Center of Evidence-based Anatomy, Sports & Orthopedic Research, Dubai United Arab Emirates

**Keywords:** Total knee arthroplasty, Patella

## Abstract

The patella remains one of the main sources of post-operative complication following total knee arthroplasty surgery. Optimal positioning of the patellar component is still a controversy with no clear-cut guidelines. Instead of choosing an empirical position, we described a novel surgical technique to better locate the patellar button based on the individual patellar anatomy of each patient.

## Introduction

Many post-operative complications can occur during a total knee arthroplasty (TKA) of which the patella is one of the main sources [1]. Patellar related complications include anterior knee pain, maltracking, subluxation or dislocation [2]. These complications are owed to the anatomy of the patient, the surgical technique and/or to the design of the patellar component [2]. The optimal position of the patellar component remains a controversy; some advocate medialization [3–6], while others support centralization over the patellar cut [7]. Due to differences in human patellar morphology as described by Wiberg [8], positioning of the patellar component would differ from one patient to another. Therefore, we have described the optimal center of the prosthetic patella (OCPP) that is dependent on the specific anatomy of every patient [9]. The aim of this article is to describe a novel surgical technique for optimal patellar component positioning during TKA based on the individual anatomy of the patient.

## Surgical technique

**Figure 1:**
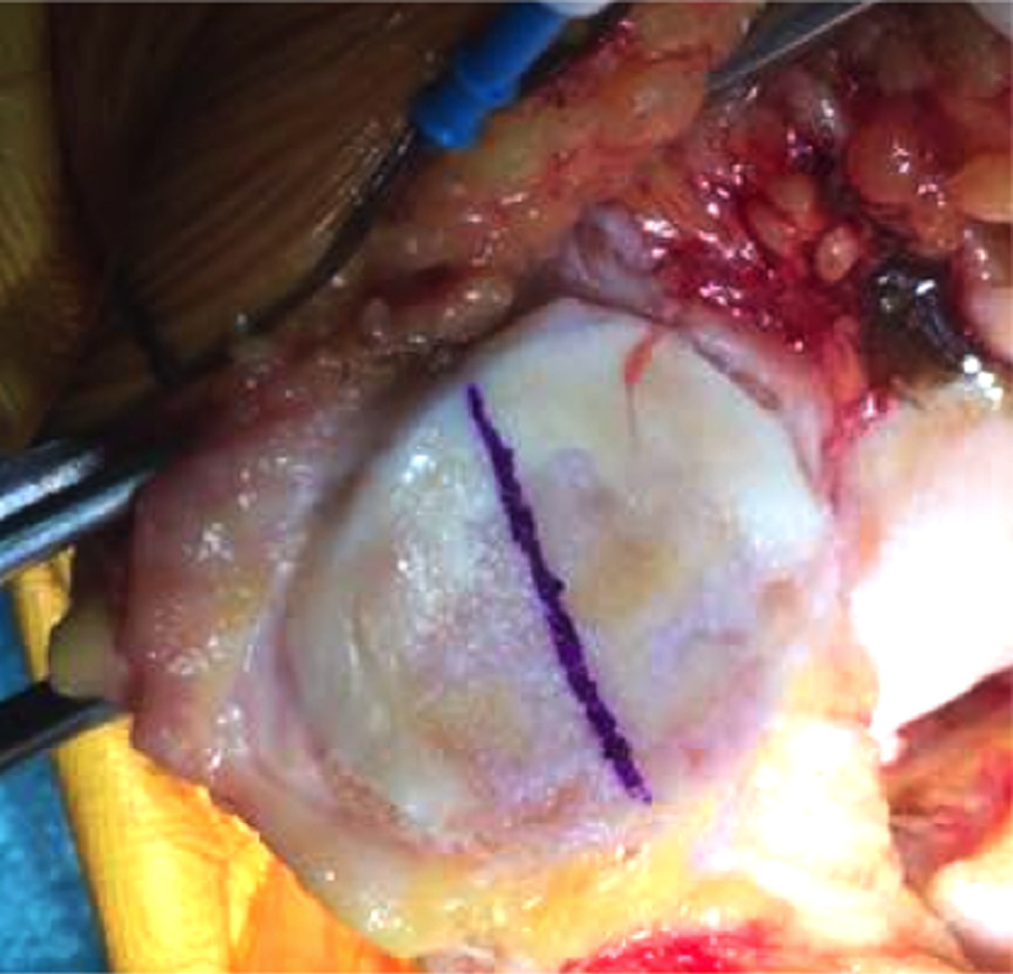
Vertical line drawn over the vertical ridge.

**Figure 2:**
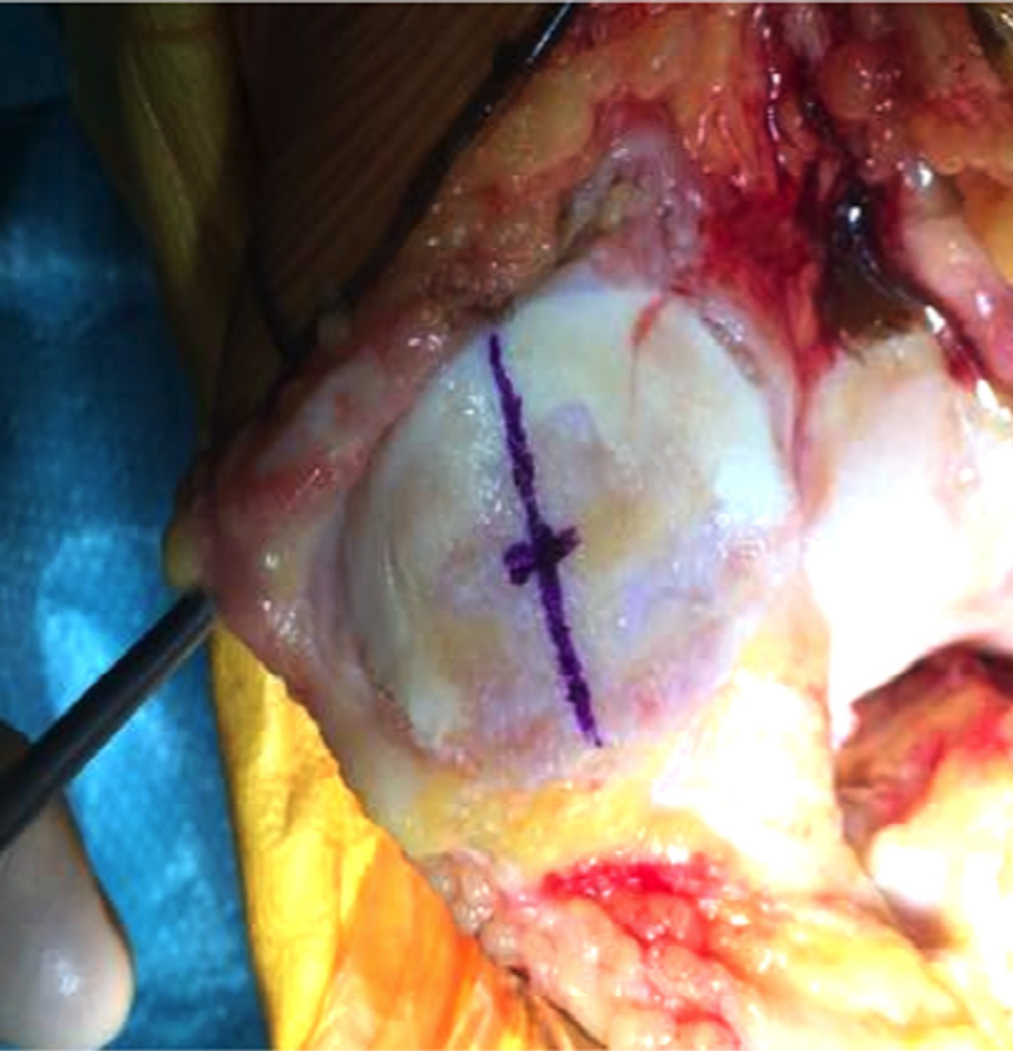
Midpoint of the vertical ridge identified as the OCPP.

**Figure 3:**
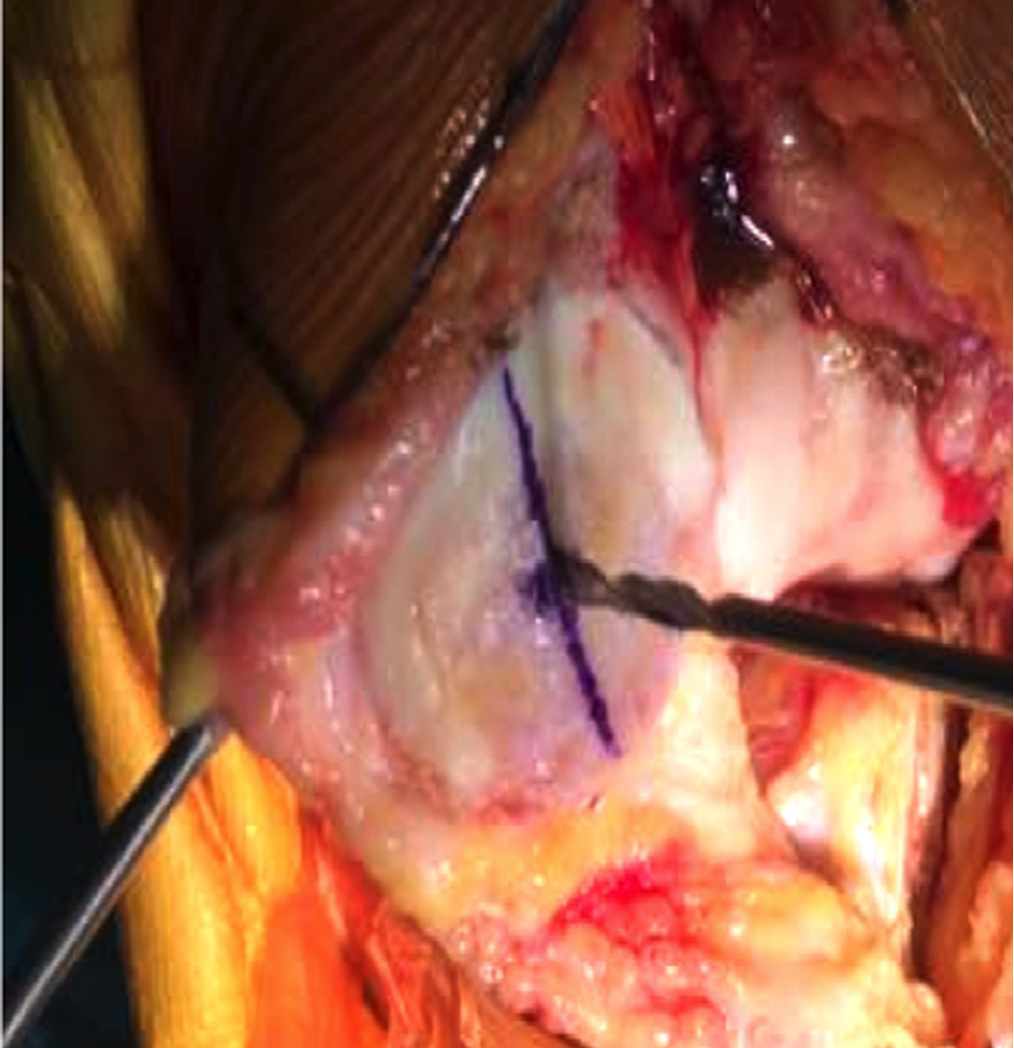
Tunnel drilled perpendicularly over OCPP.

**Figure 4:**
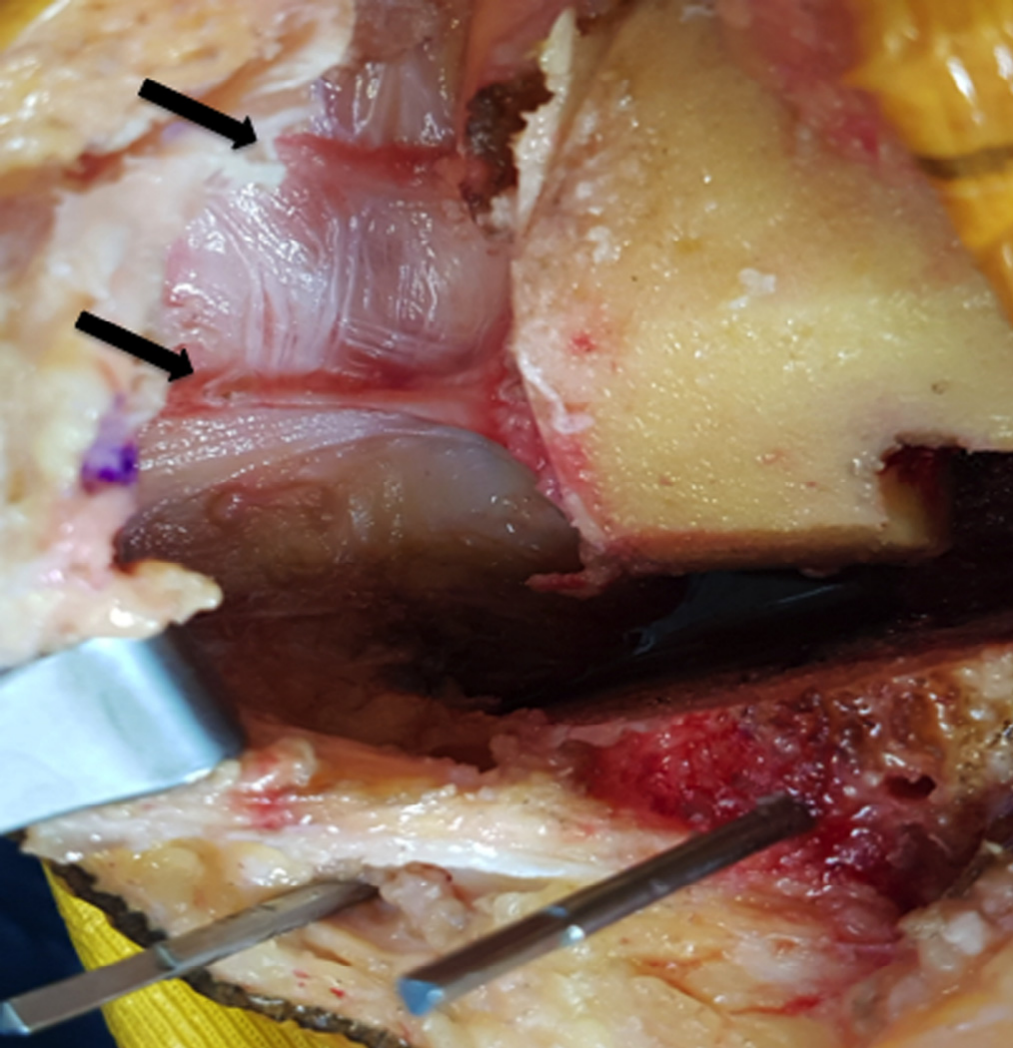
Lateral synovial folds are identified and released.

**Figure 5:**
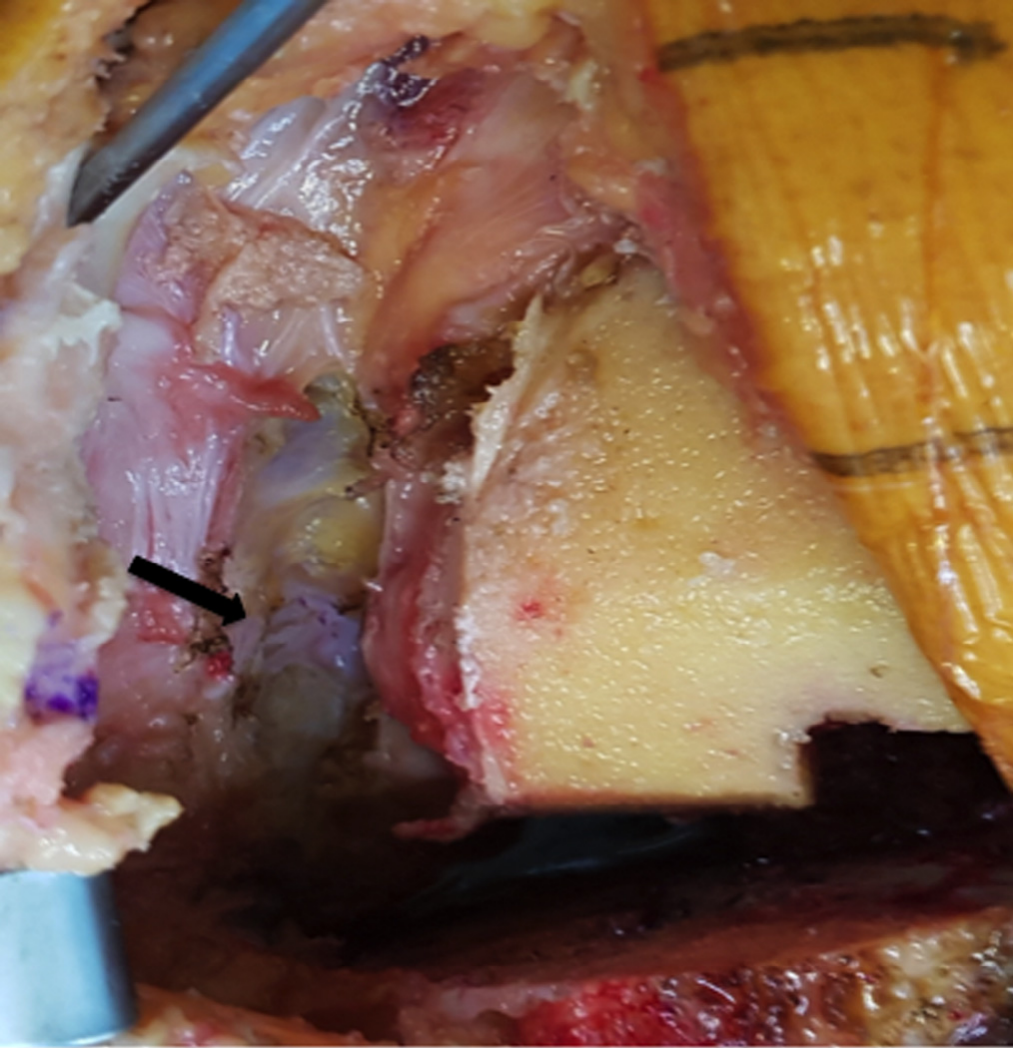
Care must be taken not to injure the lateral superior geniculate artery when performing the release.

**Figure 6:**
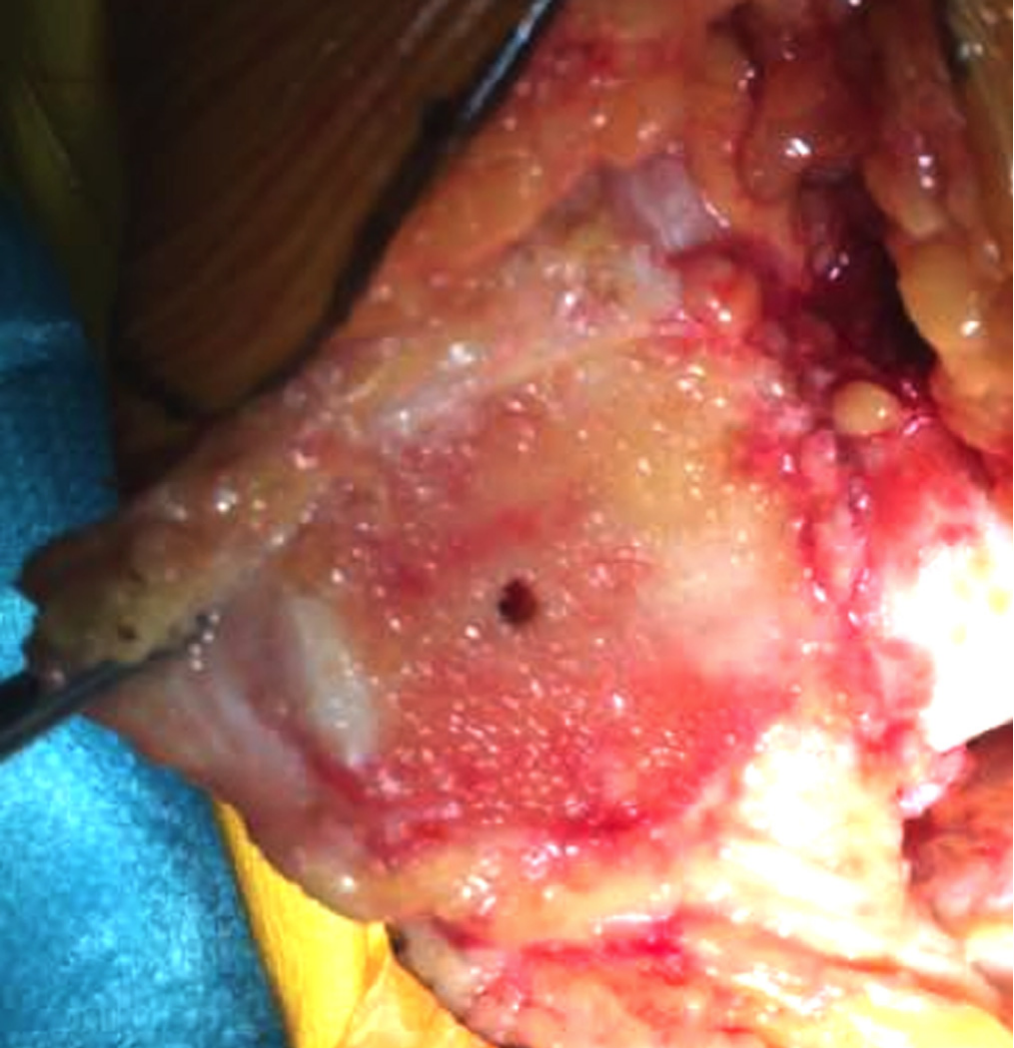
Pre-drilled hole identified on the patellar remaining cut.

**Figure 7:**
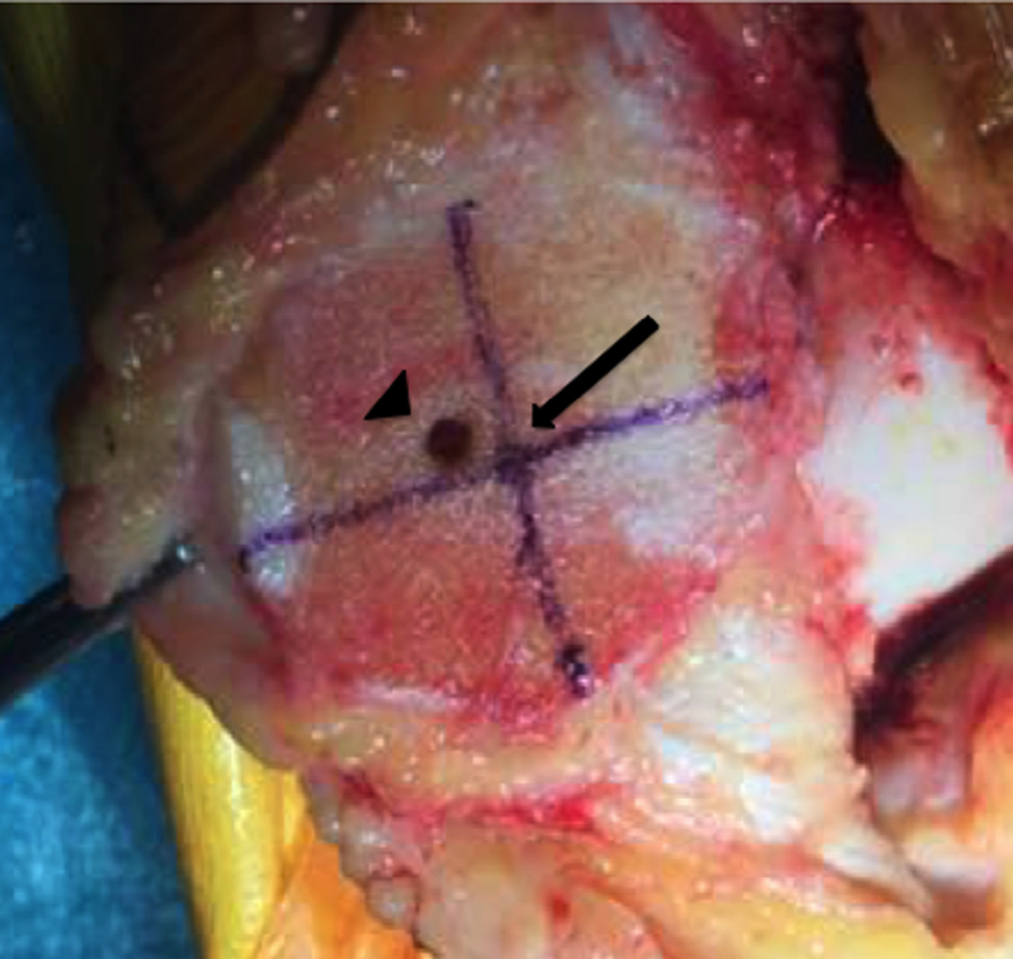
Identification of the anatomic center of the patellar cut and comparison with respect to the OCPP.

**Figure 8:**
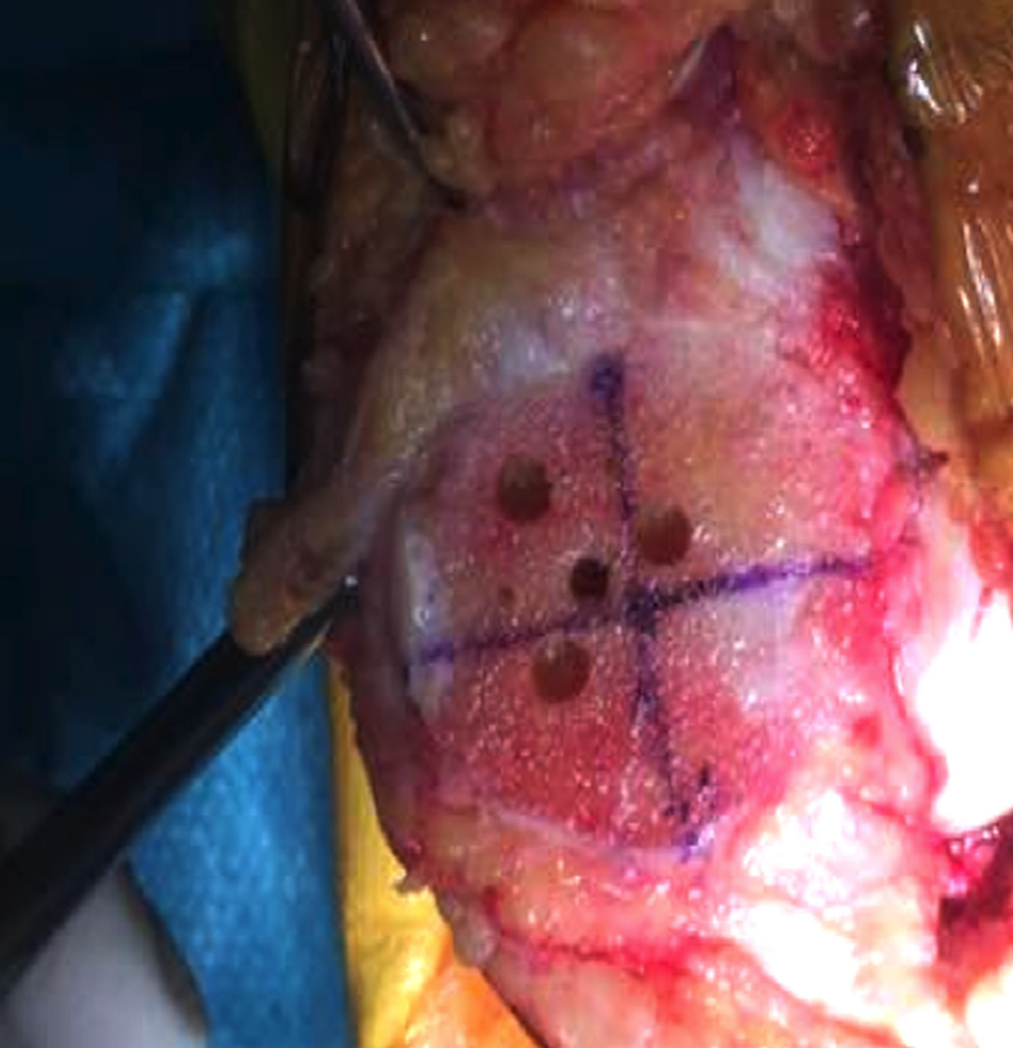
Drill guide is positioned over the OCPP and pegs are drilled.

**Figure 9:**
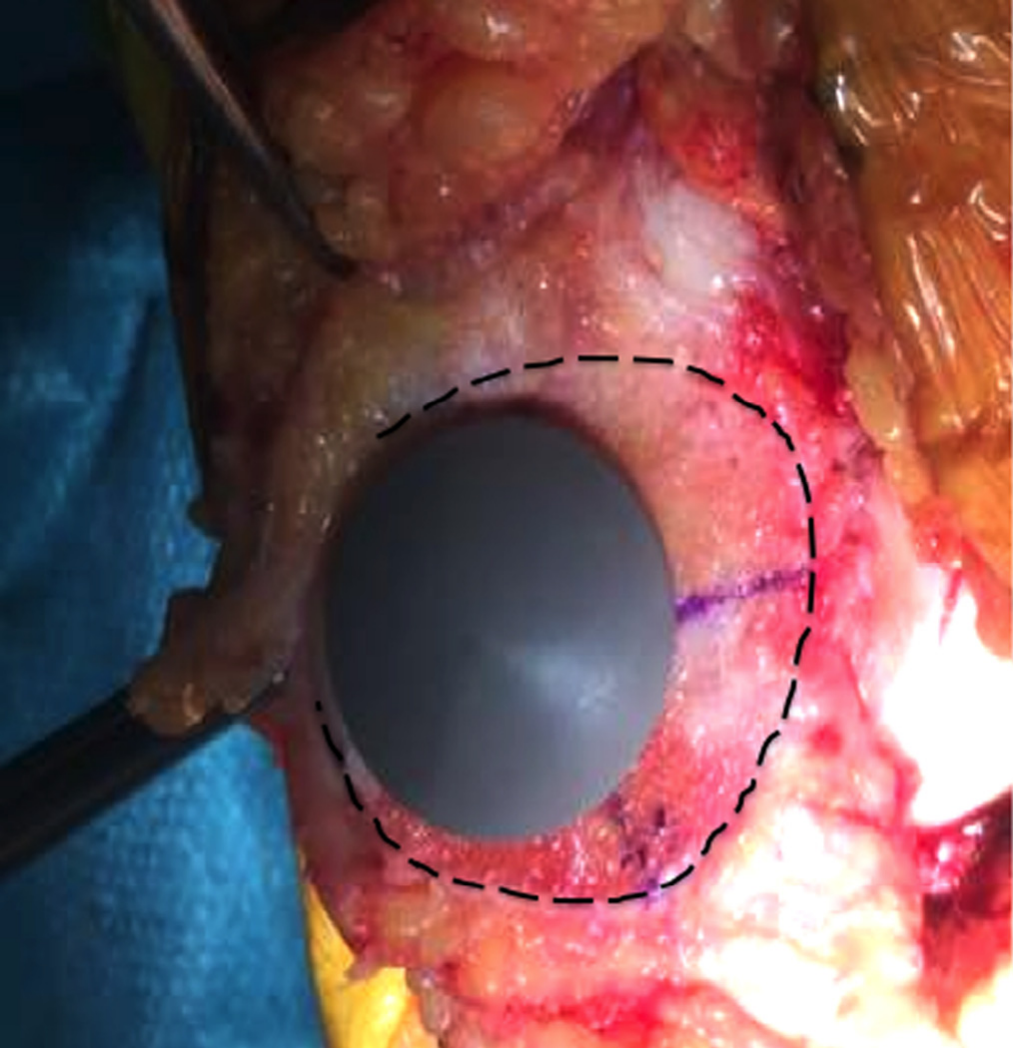
Trial component is then placed and assessed.

**Figure 10:**
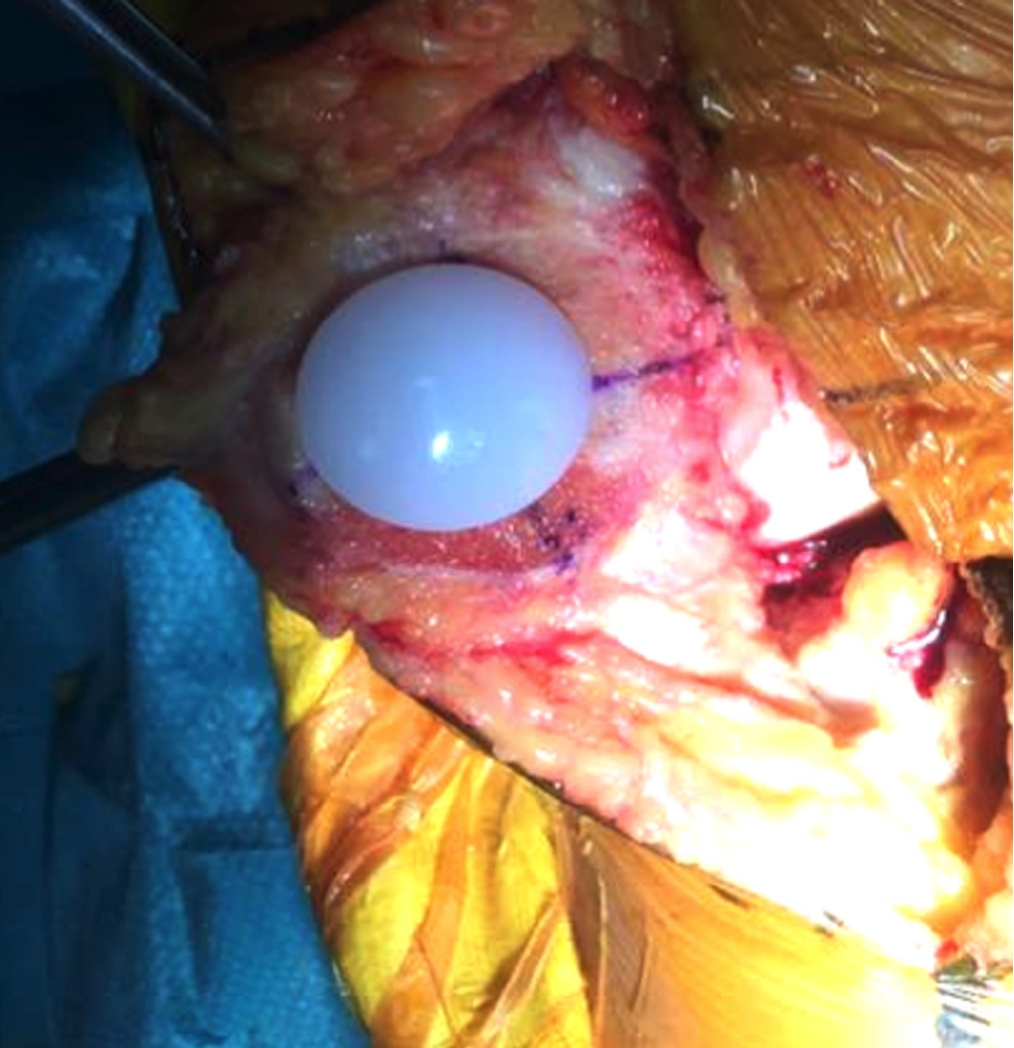
Final patellar component in place.

After prepping and draping the lower extremity, an anterior midline incision is systematically used. A medial parapatellar arthrotomy is done with lateral eversion of the patella. The tibial and femoral cuts are prepared first. The posterior aspect of the laterally everted patella is identified. Removal of any peripheral osteophytes is performed to clear the anatomic contour of the patellar cartilage. The vertical articular ridge is then identified. A vertical line is drawn from proximal to distal over this ridge (Figure 1). Using a millimeter ruler, the vertical ridge length is measured. The midpoint of the vertical ridge is identified as the OCPP (Figure 2). Using a 1.2 mm drill bit, a tunnel is perpendicularly drilled through the OCPP towards the anterior cortex of the patella without breaching it (Figure 3). The patellar cut is performed using the patellar ancillary cutting guide leaving a patellar bone thickness of 15 mm. The lateral synovial folds connecting the lateral patella to the lateral femoral condyle present in the lateral gutter are systematically released (Figure 4). Care is taken not to injure the lateral superior geniculate artery, which runs proximally (Figure 5). The pre-drilled hole is identified on the surface of the patellar remaining cut (Figure 6). We identified the anatomic center of the patellar cut in order to compare it to the OCPP (Figure 7) [9]. The patellar drill guide is centered over the OCPP and the pegs are drilled (Figure 8). The largest possible patellar component is used as long as it is centered over the OCPP without any overhanging on the medial side and superior border of the patella. The trial patellar component is then placed and tracking assessment is done (Figure 9). The final patellar component is positioned and the patellar tracking is reassessed (Figure 10).

## Discussion

We described a new surgical technique for optimal patellar component positioning during TKA. The mainstay concept of the OCPP is the identification of the midpoint of the vertical articular ridge before the patellar cut [9]. This ridge has been chosen because it is the vertical axis on which the patella articulates with the trochlear groove of the femur [10]. In addition, choosing the midline axis of the patellar cartilage will not have an impact on the patellar height as demonstrated by Caton et al. [11]. The OCPP was used in order to be as accurate as possible to reproduce the native patella-femoral joint. With this, we are able to reproduce the anatomical patellar center and function of the native patella. A previous study published by Assi et al. [9], evaluated 129 knees for patellar component position related complications. On a mean follow up of 4.5 years, there were no reports of anterior pain or patellar snapping, maltracking or subluxation. The patients noted no episodes of dislocation and no TKA revision has been planned or performed for any patellar problem.

Other studies have shown superiority of medialization [3–6] or centralization [7] of the patella empirically. These studies neither took into account the different morphologies of the patella nor the identification of a specific parameter for reproducibility. With the identification of the OCPP, the surgeon would be able to individualize the placement of the patellar component in the optimal position depending on the native patellar morphology of the patient [9].

## Conclusion

This novel surgical technique for optimal positioning of the patellar component has shown encouraging clinical results post-operatively. This technique is simple, anatomical, reproducible and cost-free.

## Conflict of interest

The authors certify having no financial conflict of interest in connection with this article.
